# Clinical Implications of Probiotics in Oral and Periodontal Health: A Comprehensive Review

**DOI:** 10.7759/cureus.51177

**Published:** 2023-12-27

**Authors:** Unnati Shirbhate, Pavan Bajaj, Manoj Chandak, Priyanka Jaiswal, Swayangprabha Sarangi, Dhwani Suchak, Lovely Bharti

**Affiliations:** 1 Department of Periodontics, Sharad Pawar Dental College, Datta Meghe Institute of Higher Education and Research, Wardha, IND; 2 Department of Conservative Dentistry and Endodontics, Sharad Pawar Dental College, Datta Meghe Institute of Higher Education and Research, Wardha, IND; 3 Department of Orthodontics, Sharad Pawar Dental College, Datta Meghe Institute of Higher Education and Research, Wardha, IND

**Keywords:** lactobacillus, sporlac, prevention, synbiotics, prebiotics, oral health, periodontitis, probiotics

## Abstract

Probiotic therapy represents a novel concept in dentistry. The microbial nature of dental plaque can be altered, or the probiotic strategy can efficiently inhibit oral pathogens. Probiotics are dietary supplements that are vital for boosting immunity as they include beneficial bacteria and yeast. In dentistry and medicine, the interest in probiotics, prebiotics, and synbiotics is increasing. By forming a biofilm and assisting in preventing dental cavities, probiotics play a crucial role in dentistry and significantly impact immunity. Prebiotics are non-digestible dietary supplements that enhance health by increasing the quantity and activity of beneficial bacteria such as Lactobacilli and Bifidobacteria. It has been demonstrated that prebiotics, in addition to probiotics, can help treat oral diseases. They promote the growth and activity of beneficial organisms while inhibiting potentially harmful bacteria's growth and activity. Synbiotics are dietary supplements that combine probiotics and prebiotics, believed to work in tandem through a process known as synergism. Studies have indicated that synbiotics, or a combination of probiotics with a prebiotic, may have greater efficacy than either supplement alone.

## Introduction and background

Bacteria have long been linked to disease and have caused much pain for humankind. Therefore, there is a mystical quality to the idea of using bacteria for health advantages. In 2003, the term "probiotics" obtained a definitive definition. Probiotics are live microorganisms that resemble the helpful bacteria, most often bacteria, found in the human intestines. They are also referred to as "good or healthy bacteria" or "friendly bacteria" [1]. Antibiotics are the antithesis of probiotics. Probiotics are bacteria, yet antibiotics kill microbes. It is this type of bacterium that sustains our health. They can boost immunity, which strengthens our defences against predators. By utilising naturally occurring helpful bacteria, which are frequently present in the oral cavities of healthy people, to provide natural protection against microorganisms damaging the oral cavity's structures, emerging probiotic technology represents a revolutionary approach to maintaining oral and periodontal health. Probiotics are dietary supplements that include specific yeasts or potentially helpful bacteria [2]. In addition to suppressing pathogens that cause and spread disease, they stimulate flora that promotes health [3].

Before they thoroughly understood the mechanisms underlying microbial metabolism and signalling, physicians and other healthcare professionals began giving intestinal bacteria, also known as probiotics, to preserve health or treat disease [3,4]. As old as microbiology itself is the idea that microbes may be used to treat or prevent illness and maintain health. Our complete knowledge of the basic needs of these organisms and the structure of the human microbiome has hindered the development of efficient probiotics. We can now construct a stronger scientific foundation for developing probiotic methods thanks to advancements in our understanding of the microbiome and its metagenome in humans and other mammals [4]. Probiotics are named after a Greek word that means "for life." The "Food and Agriculture Organisation of the United Nations (FAO)" and the "World Health Organisation (WHO)" have declared that specific kinds of probiotic strains are safe for human consumption and that there is sufficient scientific evidence to suggest that probiotic meals may have health advantages [5]. We searched articles from search engines "PubMed, Google Scholar, and Cochrane Central Register of Controlled Trials" from February 2023 using the keywords "Probiotics, synbiotics, probiotics in oral health, management, periodontal diseases." Also, we checked the articles based on the title and abstract before the full text. We have curated articles that included methodology and studies on probiotics in periodontal and oral health.

## Review

**Figure 1:**
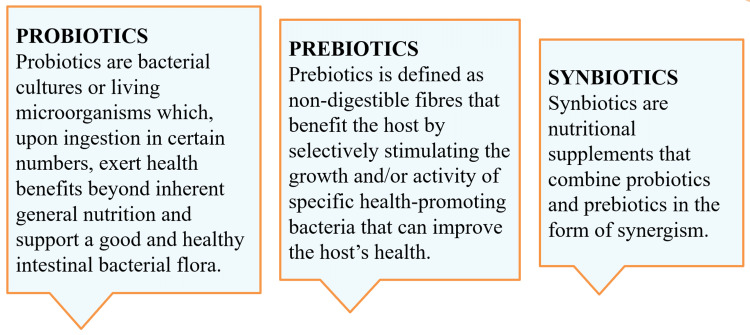
Standard definitions of Probiotics, Prebiotics, and Synbiotics.

Probiotics are live, non-pathogenic bacteria given to a host to balance its microbiome. Prebiotics and probiotics work in conjunction to produce beneficial outcomes. Gibson and Roberfroid coined the term "prebiotics" in 1995 when they employed Lactobacillus and Bifidobacterium to modify the human colon to provide health-promoting advantages [6,7]. The concept of using microbiome therapy to maintain the oral cavity's balance is relatively recent. Prebiotics are substances found in food that aid in the development or heightened activity of the host's probiotic microbes [6]. In essence, these prebiotics are indigestible fibrous substances. Conversely, symbiotics are food ingredients or dietary supplements that synergistically combine probiotics and prebiotics [7]. Figure 1 entails standard definitions of probiotics, prebiotics, and symbiotics [5-7].

Difference between antibiotics and probiotics

Vital medicines called antibiotics are used to treat bacterial infections. They end up eliminating some beneficial bacteria along with some harmful ones. This may lead to digestive issues, the most frequent of which is diarrhoea. Antibiotics are the antithesis of probiotics. Probiotics are bacteria, but antibiotics kill bacteria [8]. It is this type of bacterium that sustains our health and has the power to boost immunity, which strengthens our defences against pathogens [9]. Table 1 shows the difference between antibiotics and probiotics [8,9].

**Table 1:** Difference Between Probiotics and Antibiotics [8,9].

Probiotics	Antibiotics
Probiotics are beneficial live microorganisms.	Antibiotics target and kill or inhibit the growth of specific bacteria, causing an infection.
Have a positive influence on the microbiome.	By eliminating both good and harmful microbes, you can treat infections caused by bacteria and alter the microbiota.
They promote and maintain a healthy balance of micro-organisms in the gut.	Treat bacterial infections by eliminating harmful bacteria responsible for the disease.

History

The modern form of probiotics began more than a century ago when Russian scientist and Nobel Prize laureate Elie Metchnikoff of the Pasteur Institute in Paris discovered the breakthrough. Known as the "grandfather of modern probiotics," he is regarded as the pioneer in the subject. In India, dairy products, including buttermilk, curd, and milk, are staples of the cuisine. The dietetics literature from the Middle Ages, known as "Kshemakutuhalam," highlights the value of consuming dairy products and refers to buttermilk as the "elixir of gods." French paediatrician Henry Tissier noticed in the early 1900s that children with diarrhoea who consumed bifid bacteria with a Y shape had healthy gut flora. In 1930, "Yakult," a probiotic drink that is sold commercially and contains Lactobacillus species, was introduced in Japan. When Lilly and Stillwel used the term "probiotics" to discuss health for the first time in 1965, it went viral [7]. Researchers Haukioja et al. are credited as being among the first to examine how probiotics interact with the oral mucosa. They discussed how well lactobacilli and bifidobacterial species adhered to saliva-coated hydroxyapatite crystals [10].

Mechanism of action of probiotics

**Figure 2:**
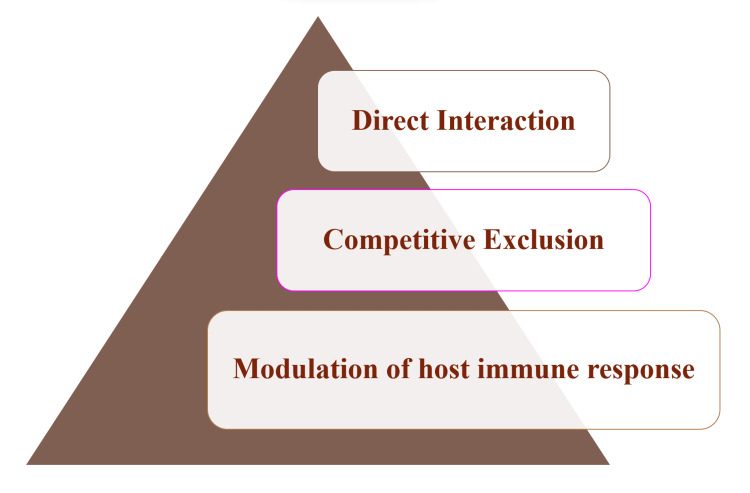
General Mechanism of Probiotics Divided Into Three Main Categories. Created by author: Unnati Shirbhate

The action of mechanisms of probiotics in the oral cavity works similarly to those in the intestine. The ecological plaque hypothesis, which postulates that selection pressure within ecological environments can alter the balance between dental disease and good dental hygiene, is consistent with the normalization of intestinal/oral microbiota. Certain bacterial species appear connected to oral health, just as some are linked to oral diseases. The three primary categories Parvez et al. identified in 2006 for the general processes of probiotics are depicted in Figure 2 [11-13].

It has been documented that certain probiotic bacteria influence both cellular and systemic immune responses. While the processes underlying immune modulation remain incompletely understood, it is known that the immune system recognises bacterial components through their interaction with specific Toll-like receptors, which in turn modulates immune responses. Specific reported Toll-like receptors have been implicated in some of these interactions. Probiotic bacteria can potentially mitigate inflammatory processes by promoting a stable microbiota and enhancing the permeability barrier of the intestine [12]. Although total secretory immunoglobulin A (sIgA) levels in saliva appear unaffected by probiotic use, oral colonisation by probiotic bacteria has traditionally been thought necessary for them to have oral benefits. However, systemic effects are complex to rule out. It is interesting to note that some probiotic strains appear to affect the nutritional content of breast milk in mothers who use them [12,13]. Regarding commensal oral microbes, several factors lend credence to the possibility of discovering bacteria that could be helpful in the treatment or prevention of oral diseases. Indeed, it has been proposed that some of the benefits of probiotics that have been seen are not unique to a few thoroughly investigated strains but rather shared by multiple species [13].

Criteria for the selection of probiotics

A prospective probiotic contender should possess various advantages when developing new strains and probiotic products. However, not all applicants will be able to meet every probiotic quality criterion. Since microbial species already present in the gut flora may have a better chance of surviving in their native environment and sustaining extreme gastrointestinal circumstances, one of the most essential factors to consider when selecting a probiotic strain is its source. One of the primary standards for determining possible probiotics is often thought to be the microorganisms' capacity to colonise the gastrointestinal tract-some of the requirements a novel probiotic candidate should meet [14]. Probiotic safety assessment must consider aspects such as pathogenicity and infectivity, intrinsic qualities, and virulence factors associated with the bacteria's toxicity and metabolic activities. Additionally, probiotics must be viable and active during their preservation and passage through the gastrointestinal system (GIT). Given that the stomach and the region around the gastrointestinal tract are the most acidic environments, it is critical to ascertain the bacterium's reaction and any modifications during this process. Another crucial selection factor is a probiotic's ability to adhere to host tissues, especially intestinal mucus and epithelial cells, to promote productive host-microbial interactions. The duration that this specific strain stays in the stomach is significantly increased by this interaction [15].

Characteristics of effective Probiotics

**Figure 3:**
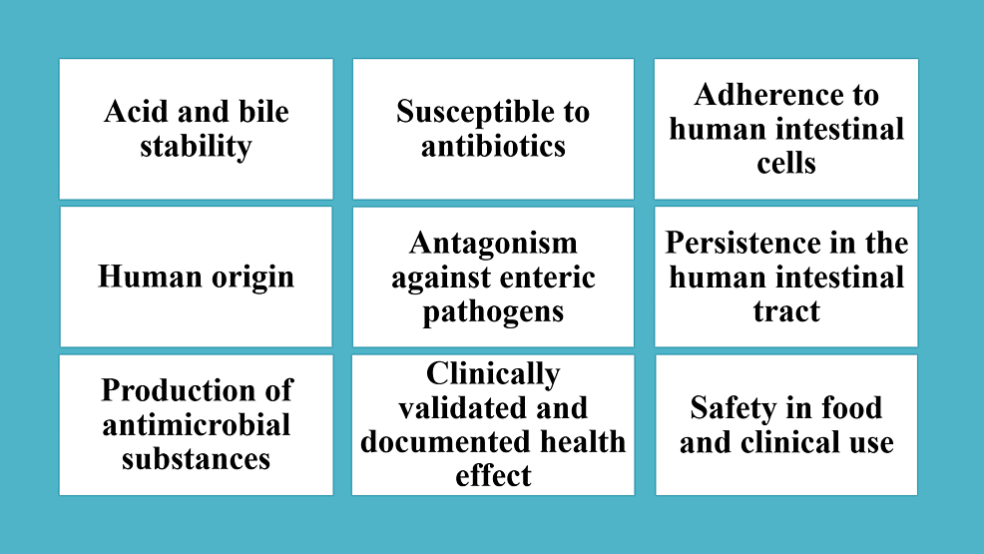
Characteristics of Effective Probiotics. Created by Author: Unnati Shirbhate

Probiotics are called "health-friendly bacteria" since they have several advantageous health qualities. Probiotics work by lowering the oral cavity's pH, preventing plaque bacteria from forming calculus and dental plaque that lead to periodontal disease. Effective probiotics often have the following characteristics: they adhere to tooth surfaces, create antimicrobial compounds that kill pathogens, reduce inflammatory processes, and change the microbial environment of the oral cavity [5,12]. The most recognised and widely used probiotics are lactobacilli [3,16]. A probiotic needs to meet several requirements to be beneficial to human oral and general health. It needs to survive in the gut environment and make it through the upper GIT to reach its site of action alive. Probiotics must be able to stay in human bile and gastric juice, adhere to epithelial surfaces, remain persistent in the human GIT, stimulate the immune system, exhibit antagonistic activity against intestinal pathogens like *Clostridium difficile*, Salmonella species, Listeria monocytogenes, and Helicobacter pylori, as well as can stabilise and modify the gut microbiota. The characteristics of efficient probiotics are listed in Figure 3 [5,12,16].

Probiotics in general health

Probiotics have demonstrated the ability to provide several health benefits, such as the avoidance of antibiotic-associated diarrhoea, which includes *Clostridium difficile*-caused diarrhoea, in the treatment of infant colic, in the management of periodontal diseases, and the induction or maintenance of remission in ulcerative colitis [17]. Beyond this, they have been shown to reduce blood cholesterol, inhibit the growth of pathogens, produce vitamin B12 and folic acid, modulate immunity, prevent cancer, treat lactose intolerance, improve intestinal microflora, and restore normal intestinal flora during antibiotic therapy [18].

Probiotics in oral health

The dental care community has become more engaged in probiotic use in the past few years, leading initiatives to lower the occurrence of oral diseases such as dental caries, periodontitis, halitosis, or infections like oral candidiasis. The genera Lactobacillus and Bifidobacterium contain the most widely utilised probiotic bacterial strains [13]. It is thought that these bacterial species constitute a typical component of the human microbiome. Lactobacilli typically account for less than 1% of the total cultivable microbiota in the oral cavity, despite the fact that no species that are specific to the oral cavity have been found. Lowering the quantity of mutans streptococci in saliva may be achieved by consuming foods that contain probiotic lactobacilli or bifidobacteria [13]. Antibiotic use appears to have significant adverse impacts, including disease resistance, alteration of the intended oral microbiota, and increased susceptibility of oral cavities to dental caries. Probiotics are used in caries treatment because their bacteria can colonise the oral cavity and fight against cariogenic microorganisms [19]. The most commonly isolated species are *Streptococcus salivarius*, *Streptococcus sanguis*, *Streptococcus mitis*, and *Streptococcus mutans*. They can colonise teeth, lower the pH in the oral cavity, and create extracellular polymer sodium (EPS) from sucrose. Preventing dental caries may be achieved by eradicating or inhibiting the proliferation of this bacterium on tooth surfaces [20]. The colloidal properties of milk appear to protect enamel because they contain both organic and inorganic chemicals that help to counteract cariogenic strains. Calcium lactate is another substrate used in dairy formulations that has anti-cariogenic qualities. In addition to its anti-cariogenic and immunomodulating properties, *Lactobacillus paracasei *has been utilised in yoghurt and probiotic products to lower salivary bacteria counts linked to dental caries in healthy adults. *Lactobacillus casei* Shirota is the single living bacterium in the popular probiotic beverage Yakult [21].

Specific individuals appear capable of allowing probiotic microorganisms such as Lactobacillus and Streptococcus to colonise their oral cavity while using products that contain them. For a brief period following use, the oral cavity becomes temporarily occupied with *Streptococcus salivarius *K12, a medication used to treat halitosis or oral malodor [13,22]. Probiotics are available as treatments for halitosis associated with the gut and mouth. Halitosis is not a disease; instead, it is an awkwardness. While some oral conditions, such as periodontitis, may be the underlying reason, the oral cavity is responsible for 90% of cases [23]. Gargling with a Weissella cibaria solution decreased the production of hydrogen sulphide and, as a result, a decrease in bad breath or halitosis [24]. According to De Boever and Loesch, halitosis is primarily caused by the proteolytic bacteria colonising the tongue, which breaks down dietary proteins [25]. Burton et al. investigated the use of *Streptococcus salivarius *K12 (SS K12) tablets and placebo and probiotics chlorhexidine mouth rinses in treating halitosis, showing a substantial decrease in halitosis levels [26].

Cariogenic mutans streptococci strains may be produced and proliferate more quickly in an ecological environment facilitated by the intricate design of orthodontic bands and brackets. In 2009, Cildir et al. carried out a probiotic-based study. They discovered that orthodontic patients using fixed appliances might have lower salivary levels of mutans streptococci if they regularly consumed fruit yoghurt containing *Bifidobacterium animalis *subspecies Lactis DN -173010 [27]. Patients in The Netherlands have reported anecdotally that eating buttermilk, which contains *Lactococcus lactis* species and *Lactococcus cremoris*, which can create antimycotics and other chemicals, may improve the life of indwelling voice prosthesis. According to recent research, consuming 2 kg of Turkish yoghurt per day efficiently removes biofilm growth on indwelling voice prosthesis; this effect may be attributed to the yoghurt's inclusion of *Lactobacillus bulgaricus* and *Streptococcus thermophilus *[24].

Probiotics in periodontics

Inflammation of the periodontal tissues - the gingiva, the alveolar bone socket, the tooth's outer layer of roots, and the connective tissue around them - is known as periodontal disease. Plaque buildup is the first manifestation of this disease. Bleeding on probing, colour changes, swelling, pain, and, in more advanced stages, tooth mobility are all signs of periodontal disease [28]. By lowering salivary pH and generating antioxidants that use free electrons required for plaque mineralisation, probiotics inhibit the growth of carcinogenic bacteria, preventing plaque formation. Probiotics, therefore, protect against periodontal disease in this manner. Numerous studies have documented positive outcomes from probiotic use in treating periodontitis, gingivitis, plaque accumulation, and a notable decrease in periodontopathogens [8]. Probiotics are increasingly being used in the maintenance and treatment of periodontal disease. Probiotics effectively inhibit periodontopathogens and biochemical markers associated with inflammation, such as interleukin (IL)-1β, matrix metalloproteinase (MMP)-8, and tissue inhibitor of metalloproteinase (TIMP)-1, in addition to improving indices of periodontal health, bleeding on probing (BOP), probing pocket depth (PPD), clinical attachment level (CAL), and gingival crevicular fluid (GCF) volume. *Porphyromonas gingivalis*, *Aggregatibacter actinomycetemcomitans*, *Tannerella forsythia*, and *Treponema denticola *are the main periopathogens. *Streptococcus oralis *and *Streptococcus uberis *have been shown to inhibit the spread of infections in both animal and laboratory models [29].

Periodontal tissues become more vulnerable to various periodontal diseases when these bacteria are absent. A chewing gum called "PerioBalance" is made especially to combat periodontal disease. It is a combination of two *Lactobacillus reuteri *(*L. reuteri*) strains chosen for their ability to work in harmony to fight cariogenic bacteria. *L. reuteri *was assessed by Krause et al. in individuals with recurrent gingivitis. Patients with gingivitis ranging from moderate to severe were recruited. *L. reuteri *strains were given in addition to scaling and root planing (SRP) of the tooth's surface. The group that chewed probiotic chewing gums demonstrated benefits in their clinical markers after two weeks [30]. Probiotics come in various forms used to treat periodontal diseases, including tablets, lozenges, toothpaste, and mouthwash. While probiotics in toothpaste and mouthwash are frequently utilised in periodontal maintenance, probiotics in periodontal therapy are typically manufactured into tablets [29,30].

Using certain beneficial bacteria in addition to SRP prevents periodontopathogens from reoccupying periodontal pockets and preserves the epithelial barrier by maintaining the expression of tight junction proteins. "Gum PerioBalance" is said to have a balancing impact on oral microbiota and to enhance oral health. Probiotic mouthwashes have antiplaque qualities that reduce the adherence of bacteria to the tooth surface, inhibit the growth and proliferation of microorganisms on the tooth surface, prevent the development of intercellular plaque matrix, change the biology of plaque to reduce the production of cytotoxic products, and change the ecology of plaque to include a less pathogenic flora [28-30].

Commonly used probiotics

Probiotics are live, beneficial microorganisms that offer the host several health benefits. Spore-forming Bacillus species have been utilised in probiotic form for the past fifty years. A probiotic supplement called Sporlac powder contains strains of healthy *Lactobacillus sporogenes*. Typical commercially available probiotics are enlisted in Table 2 [31].

**Table 2:** Common Commercially Available Probiotics and Their Dosage Forms. [31]

Commercially available probiotics	Dosage form
BACIPRO® Unique Biotech	Oral suspension, capsules, dietary supplement
ENTEROGERMINA® Sanofi	Oral suspension, capsules
TUFPRO® Unique Biotech	Oral suspension
BENEGUT® Abbott	Oral suspension
PROALANA-B® Sparlife	Oral suspension
PROCILLUS® Du Pont nutrition	Oro dispersible granules

Administration of Probiotics

Probiotic-supplemented dairy products are an easy way to incorporate probiotics into dietary regimens because they are a natural oral means of administration. However, specially designed applications, formulas, devices, or carriers with gradual release of probiotics may be required to prevent or treat oral diseases. Montalto et al. gave a Probiotic mix in both liquid and capsules in 2004; no statistically significant difference was observed. The effectiveness of *L. reuteri* in reducing the amount of *Streptococcus mutans *has also been compared to two non-dairy administration methods: a lozenge and a Life-top straw [32]. A specifically constructed straw with a reservoir retaining probiotics has also been demonstrated. When compared to those who received a placebo, half of the patients had significantly lower salivary *Streptococcus mutans *levels when administered by either of the two methods. Chewing gum with *L. reuteri* Prodentis is a recent innovation for caries prophylaxis. This was advertised to control the amount of Streptococcus mutans in the oral cavity when taken twice daily [30,32].

Safety Concerns

An essential phase in selecting and assessing probiotics is the safety evaluation procedure. The most extensive evidence of the safety of many probiotics may come from their lengthy history of safe use; nonetheless, only a few probiotic strains have undergone rigorous safety testing. Even though a small number of lactobacilli and bifidobacteria have been linked to bacteremia in rare cases, usually in patients with severe underlying conditions, the safety of these genera's members is widely acknowledged because of their long history of safe usage and lack of toxicity [12,32].

Features of bacteria, as well as strain and genus safety attributes, are considered general safety aspects. The stability and safety of probiotics are affected by a number of parameters, including adhesion, invasive potential, resistance to low pH, pancreatic juice, bile acid, gastric juice, colonisation, and in vivo survival. Functional and physiological aspects of safety to be considered include adherence to the intestinal epithelium or tissue virulence, antagonistic activity against pathogens, immune response stimulation or suppression, selective stimulation of beneficial bacteria and suppression of harmful bacteria, and clinical side effects to volunteers or patients [33,34].

Limitations

Probiotic therapy carries various risks besides the many benefits and health advantages of probiotics and probiotic dietary items. These dangers mainly relate to safety in susceptible populations, such as immunocompromised individuals (older adults, pregnant women, and infants), those with serious illnesses, and hospitalised patients. Probiotics can directly affect the host in addition to interacting with commensal microorganisms. Future research faces several significant challenges, including understanding these relationships. Other considerable hurdles are understanding their mechanism, defining the intake levels required to produce such effects, and, more precisely, identifying which probiotic strains can provide specific health advantages [35-37].

Future Aspects

Because probiotics and prebiotics affect animal immunity, gut microbiota, dietary intake, and productivity, they may find more significant usage in the future as supplemental additives that promote growth and improve health. A novel approach to preventing dental caries is probiotic treatment. Probiotics' ability to inhibit cariogenic streptococci may help prevent dental caries in children [19]. As interest in modifying the microbial markers of health and illness rises, probiotics and prebiotics are starting to be influenced by personalised nutrition and precision medicine. Innovation in quality assurance approaches to evaluate dosage, viability, and structural and functional integrity is driven by the need for probiotics across various product forms [38].

## Conclusions

Probiotics have been shown to enhance dental health, creating new avenues for understanding the relationship between diet and oral health. They modify the immune response of the host or the balance of the gastrointestinal (GI) microbiota to treat or prevent disease. Probiotics have a lot of beneficial effects that improve dental health, providing more alternatives for treating and preventing oral disorders. With the advent of probiotics, they can be used wisely to support various treatment strategies and help preserve dental, periodontal, and overall health. The optimal probiotic strains and the best ways to administer them for oral health issues require well-established clinical trials and investigations.
